# Pathogenic *NF1* truncating mutation and copy number alterations in a dedifferentiated liposarcoma with multiple lung metastasis: a case report

**DOI:** 10.1186/s12881-020-01137-4

**Published:** 2020-10-12

**Authors:** Yoon-Seob Kim, Sun Shin, Seung-Hyun Jung, Yeun-Jun Chung

**Affiliations:** 1 Department of Microbiology, College of Medicine, The Catholic University of Korea, Seoul, Republic of Korea; 2 Integrated Research Center for Genome Polymorphism, College of Medicine, The Catholic University of Korea, Seoul, Republic of Korea; 3 Precision Medicine Research Center, College of Medicine, The Catholic University of Korea, Seoul, Republic of Korea; 4 Department of Biochemistry, The Catholic University of Korea, Seoul, Republic of Korea; 5 Biomedicine & Health Sciences, The Catholic University of Korea, Seoul, Republic of Korea

**Keywords:** Case report, Copy number alternation, Liposarcoma, Mutation, NF1, Copy number alteration

## Abstract

**Background:**

Dedifferentiated liposarcoma (DDLPS), which accounts for an estimated 15–20% of liposarcomas, is a high-grade and aggressive malignant neoplasm, exhibiting a poor response to available therapeutic agents. However, genetic alteration profiles of DDLPS as well as the role of *NF1* mutations have not been studied extensively.

**Case presentation:**

The current study reports a patient presenting with rapidly growing DDLPS accompanied by multiple lung and pleural metastases, in whom whole-exome sequencing revealed a *NF1* truncating mutation of the known pathogenic variant, c.C7486T, p.R2496X, as well as multiple copy number alterations (CNAs), including the well-known 12q13–15 amplification, and multiple chromothripsis events encompassing potential cancer-related genes.

**Conclusions:**

Our results suggest that, in addition to the 12q13–15 amplification, *NF1* inactivation mutation and other CNAs may contribute to DDLPS tumorigenesis accompanied by aggressive clinical features.

## Background

Liposarcoma is the most common type of adult soft tissue sarcoma. Dedifferentiated liposarcoma (DDLPS), which accounts for an estimated 15–20% of liposarcomas, is a high-grade, aggressive disease that shows a poor response to available therapies [1]. Genetic analysis of DDLPS via The Cancer Genome Atlas (TCGA) project revealed that, compared with other solid tumors, DDLPS carried frequent copy number alterations (CNA) including recurrent amplification at 12q13–15, and relatively lower somatic mutations with only a few recurrently mutated genes, such as *TP53* and *ATRX* [2]. Regarding CNAs, DDLPS is characterized by highly recurrent amplifications in the 12q13–15 region that contains the potential oncogenes, *MDM2*, *CDK4*, *YEATS4*, and *FRS2*, and the adipocytic differentiation factors, *DDIT*, *PTPRQ*, and *HMGA2* [2–6]. In addition to the 12q13–15 amplification, other CNAs such as the gain of 1p32 (*JUN*), and the loss of 17q11 (*NF1*) and Xq21 (*ATRX*) have been identified in DDLPS [2–6]. However, genetic alteration profiles of DDLPS have not been studied extensively.

According to studies such as GENIE [7] and TCGA [2], *NF1* mutations in DDLPS are rare, being limited to 2/179 cases in GENIE and 1/56 cases in TCGA. (Fig. S1A). *MDM4* amplification was observed in 0.6 and 5% of DDLPS cases via the GENIE study [7] and the TCGA study [2], respectively (Fig. S1A). In TCGA soft tissue sarcoma project (*N* = 265) in cBioPortal database, patients carrying *NF1* truncating or missense mutations showed significantly lower *NF1* mRNA expression levels than those in non-mutated cases. (Mann-Whitney U test, *P* = 0.012) (Fig. S1B). Patients carrying *MDM4* gains or amplifications showed significantly higher *MDM4* mRNA expression levels compared with those of diploid or shallow deletion cases. (Mann-Whitney U test, *P* < 0.001) (Fig. S1C).

Herein, we report a patient with rapidly growing DDLPS with multiple lung and pleural metastasis, in whom whole-exome sequencing (WES) revealed a *NF1* truncating mutation of a known pathogenic variant and multiple CNAs including *MDM4* gain.

## Case presentation

**Fig. 1:**
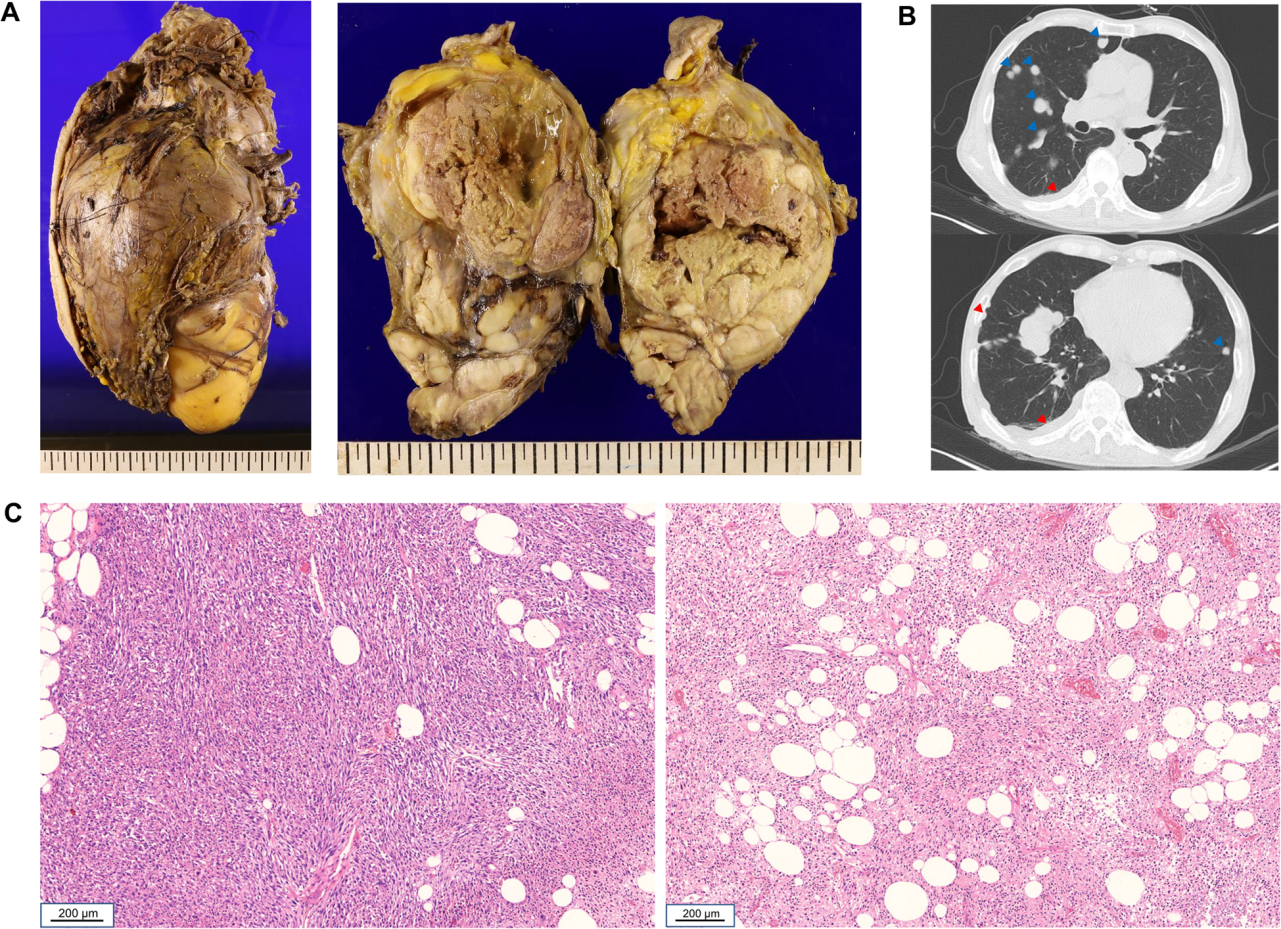
Pathological and radiological features of the DDLPS case. **a** On gross pathology, tumor showed well circumscribed tumor mass with internal multilobulated areas. **b** Multiple lung (blue arrow) and pleural metastases (red arrow) were detected by chest computed tomography. **c** Histological findings of representative tumor area showed dedifferentiated areas with spindle cells without lipogenic portion (left panel) and less dedifferentiated areas with round cells with lipogenic portion (right panel) (hematoxylin and eosin, original magnification, × 100)

An eighty four-year old male visited the outpatient clinic complaining of a painful mass that had been present on his left thigh for 3 years. He was medically healthy and did not have a previous medical or familial history of malignancy. There was no clinical sign of neurofibromatosis. He had twice undergone excisional biopsies (18 and 3 years ago) of the mass at the same location under the presumed diagnosis of lipoma. Preoperative magnetic resonance imaging revealed an approximately 17.5 × 16.4 × 30.2 cm sized, extensive, lobulated heterogeneous mass with T2 high, T1 high signal intensity involving the left thigh. A wide local excision was performed. Gross pathology of the tumor showed a well circumscribed tumor mass with internal multi-lobulated areas (Fig. 1a). Multiple lung and pleural metastases were diagnosed via chest computed tomography (Fig. 1b). Histological findings showed dedifferentiated areas with spindle cells without a lipomatous portion and less dedifferentiated areas consisting of round cells with lipomatous portions, along with infiltrated polymorphonuclear cells (Fig. 1c). Based on these clinical and pathological findings, the mass was diagnosed as DDLPS. The patient died 2 months following surgery.

A DDLPS frozen tissue was obtained from the biobank of Seoul St. Mary Hospital (Seoul, Republic of Korea). Genomic DNA was extracted by microdissection of tumor cell rich area (> 70% of tumor cell purity) and whole blood of the patient using the DNeasy Blood & Tissue Kit (Qiagen, Hilden, Germany). WES was performed using the Agilent SureSelect Human All Exome 50 Mb Kit (Agilent Technologies, Santa Clara, CA) and Illumina HiSeq 2500 platform (Illumina, San Diego, CA). Data pre-processing was done using the best practices workflows of The Genome Analysis toolkit (GATK, v4.1.1) (https://software.broadinstitute.org/gatk/) to align the sequence reads with the human reference genome (UCSC hg19) and local realignments with base recalibration, and to identify somatic mutations. The web ANNOVAR package was used to select somatic mutations located in the exonic sequences and to predict their functional consequences [8]. In order to obtain reliable and robust mutation calling, the following variants were eliminated: (i) read depth fewer than 20 in either the tumor or matched constitutional tissues; (ii) polymorphisms listed in the population databases of East Asians with a minor allele frequency 0.1% or more; and (iii) variant allele frequencies less than 5%. Catalogue of Somatic Mutations in Cancer (COSMIC) mutation signatures were obtained via a Mutalisk package [9] using known mutation signatures of soft-tissue sarcoma [2] (signature 1, 2, 5, and 13). To define CNAs, we used the ngCGH module and SNPRank Segmentation statistical algorithm in NEXUS software 9.0 (Biodiscovery, El Segundo, CA). Segments were classified as gains or losses when the log2 ratio was greater than 0.25 or less than − 0.25, respectively. Amplification was defined as a log2 ratio greater than 1.0.

**Fig. 2:**
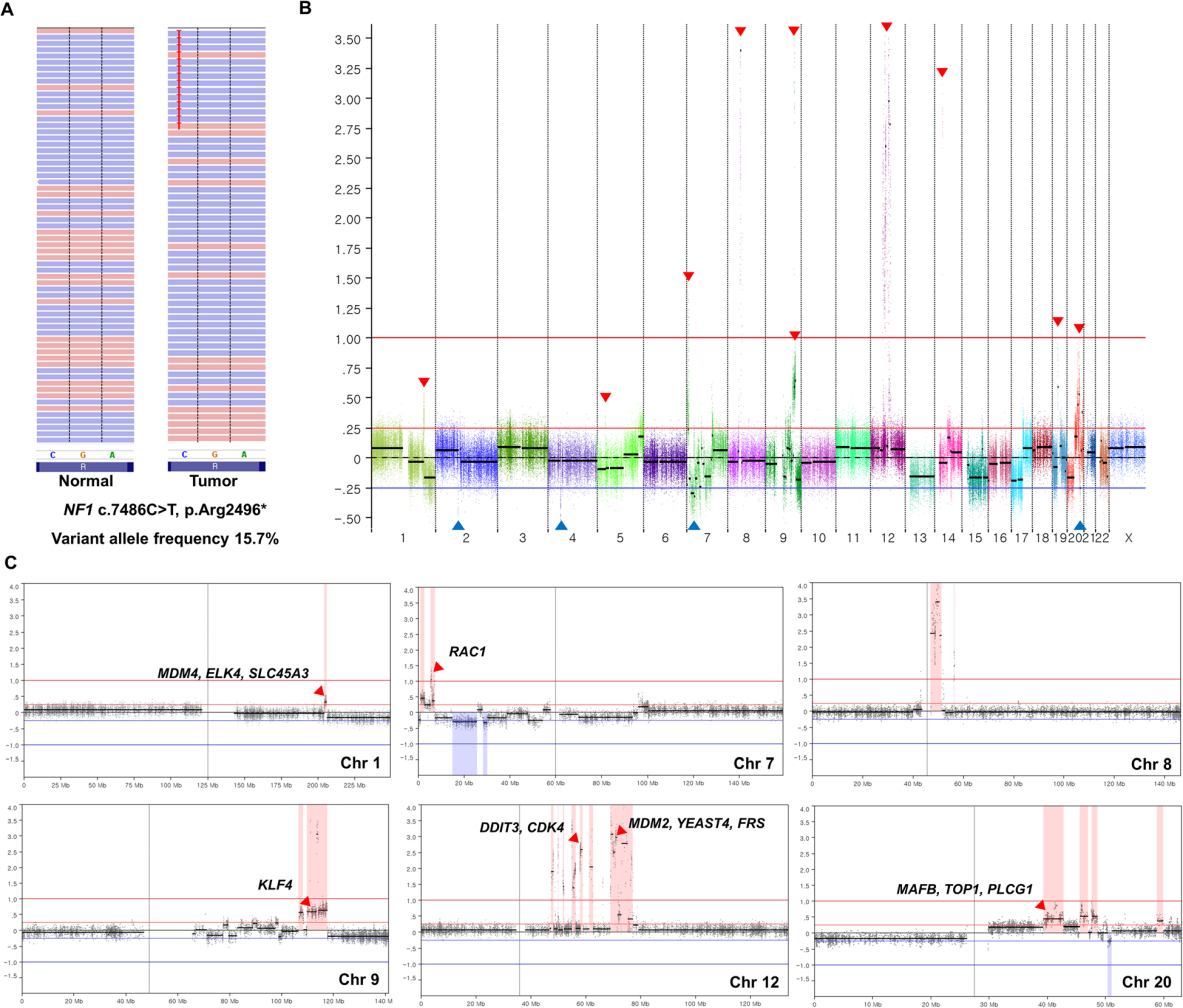
Identification of genomic alterations by WES from a DDLPS patient. **a** Identification of a somatic *NF1* mutation. The red T letters indicate the presence of a truncating mutation (c.7486C > T, p.Arg2496*, variant allele frequency 15.7%) in *NF1* gene. **b** Genome-wide copy number alternation profile of the DDLPS patient. X-axis represents individual chromosome, and Y-axis represents depth ratio (tumor/constitutional) in log2 scale. Red and blue lines indicate the threshold of copy gain and copy loss. Bold red line indicates the threshold of amplification. Red and blue arrows represent the copy gain/amplification and loss, respectively. **c** Copy number alternation profiles of chromosome 1, 7, 8, 9, 12, and 20. X-axis represents individual chromosome, and Y-axis represents depth ratio (tumor/constitutional) in log2 scale. Red arrows represent the copy gain or amplification regions where known DDLPS related genes are located. Chromothripsis events were observed in 7p, 9q, and 20q

The average sequencing depths for tumor and constitutional DNA were 218X and 223X, respectively (Table S1). A total of 36 non-silent mutations were identified in the exonic area (Table S2), which corresponded to a mutation rate of 0.73 per Mb. The *NF1* stop gain mutation (c.7486C > T, p.Arg2496*, variant allele frequency 15.7%) was identified among the cancer-related genes listed in the Cancer Gene Census of COSMIC database (Fig. 2a). This variant was classified as ‘pathogenic’ in ClinVar DB (RCV000218957.1), and not reported in population level variant databases. There is no germline variant classified as ‘pathogenic’ or ‘likely pathogenic’ in ClinVar DB. Thirty-five regions affected by CNAs were found, which correspond to 2% of the genome, including amplifications on 12q13–15, which included known genes associated with DDLPS pathogenesis, such as *DDIT3*, *CDK4*, *MDM2*, *YEATS4*, and *FRS* [2, 4–6] (Figs. 2b & c; Table S3). In addition to 12q13–15 amplifications, copy gains or amplifications on 1q32, 5p13, 7p22, 8q11–12, 9q31, 12q15–21, 14q12, 19p12–13, and 20q12–13 as well as copy losses on 2p11, 4p11-4q11, 7p21-p14, and 20q13.2 were observed. Several potential cancer-related genes, such as *MDM4, ELK4, SLC45A3, RAC1, KLF4, MAFB, TOP1, PLCG1*, and *PTPRT,* were located in the copy gain/amplification regions (Fig. 2c; Table S3). Chromothripsis events were also observed in 7p, 9q, and 20q (Fig. 2c). In a mutational context, C > T mutations were predominant and signature decomposition analysis indicated predominant signatures 1 and 5 (clock-like mutational process) followed by a minor proportion of signatures 2 and 13 (APOBEC-related), which was consistent with the results of a TCGA study on DDLPS [2] (Fig. S2).

## Discussion and conclusions

Although genetic alteration of *NF1* is commonly found in liposarcomas (10–20%) [5, 6], inactivation of *NF1* by a mutation or a deletion may contribute to the aggressiveness of liposarcoma [5, 10]. Processes associated with the occurrence of *NF1* mutations in DDLPS remain unclear. Using WES, we identified a pathogenic *NF1* truncating mutation with multiple CNAs in a DDLPS case exhibiting aggressive clinical features. The *NF1* truncating mutation identified in this case was classified as a ‘pathogenic’ event (ClinVar) which could act as a driver. In spite of the tumor being located in the extremities, a relatively favorable area [11], the patient presented with aggressive features of a rapidly growing tumor mass accompanied by multiple lung and pleural metastases.

Decreased *NF1* expression may lead to dysregulation of the RAS/MAPK pathway, thus contributing to tumorigenesis of the sarcoma [12]. In addition to the known 12q13–15 amplification, this case revealed other CNAs where potential cancer related genes, such as *MDM4*, are located. Amplification of *MDM4* (1q32.1) is known to play a synergistic role by inducing the inactivation of *TP53* and amplification of *MDM2* [13]. Pissaloux et al. reported that a subset of DDLPS exhibited *MDM4* amplification as an oncogenic alternative to *MDM2* amplification [14]. Chromothripsis events were observed in 7p, 9q, and 20q where potential cancer related genes, such as *RAC1, KLF4, MAFB, TOP1, PLCG1*, and *PTPRT*, are located. This result was compatible with that of a previous study which reported that 100% of liposarcomas (18/18) showed chromothripsis [15].

In conclusion, we report a DDLPS patient who presented with aggressive clinical features. The patient harbored *NF1* truncating mutations with multiple CNAs, including the well-known 12q13–15 amplification, and multiple chromothripsis events. Further studies may be needed to elucidate the role of *NF1* inactivation mutations and multiple CNAs in DDLPS tumorigenesis accompanied by aggressive clinical features.

## Supplementary information

**Additional file 1.**

## Data Availability

WES data were deposited in the SRA database (Project ID: PRJNA529696). Human reference genome used in this study (UCSC hg19) can be downloaded at the following url: https://hgdownload.soe.ucsc.edu/downloads.html. Accession numbers of the putative pathogenic variant in this study (NF1 c.7486C > T, p.Arg2496*) in ClinVar database is RCV000218957.1. Functional annotation of genetic variants listed in Supplementary Table S2 is generated by the web ANNOVAR package available at the following url: http://wannovar.wglab.org/. Genes listed in The Cancer Gene Census tier 1 curated by COSMIC project (v91 release) is available at the following url: https://cancer.sanger.ac.uk/census. All other remaining data are available within the article and in the supplementary data, or available from the authors upon reasonable request.

## Abbreviations

CNA: Copy number alterations
DDLPS: Dedifferentiated liposarcoma
TCGA: The Cancer Genome Atlas
WES: Whole exome sequencing
