# Supplementary material for: Pathogenic NF1 truncating mutation and copy number alterations in a dedifferentiated liposarcoma with multiple lung metastasis: a case report
Source: BMC Med Genet. 2020 Oct 12;21:200. doi: 10.1186/s12881-020-01137-4 (PMC7552537; doi:10.1186/s12881-020-01137-4)
Supplement: Supplementary file 1 — Additional file 1. [file 12881_2020_1137_MOESM1_ESM.docx]

Supplementary Table S1. Description of whole exome sequencing data

| Sample | Sequencing reads | Mapped reads (%) | Mapped reads  in exon (%) | Mapping quality (mean) | Coverage  (mean) | % of bases  (>=20 reads) |
| --- | --- | --- | --- | --- | --- | --- |
| Normal | 188,731,104 | 188,481,374 (99.87%) | 130,470,135 (69.13%) | 48.3 | 223 | 99.05% |
| Tumor | 187,689,817 | 187,465,949 (99.88%) | 127,873,108 (68.13%) | 48.3 | 218 | 98.97% |

Supplementary Table S2. Somatic non-silent point mutations and indels

| Gene | Chr | Position | Ref | Alt | Amino acid change | Exonic function | VAF* | Cancer gene census** | ClinVar DB | COSMIC variant | Polyphen2  prediction*** |
| --- | --- | --- | --- | --- | --- | --- | --- | --- | --- | --- | --- |
| PADI1 | chr1 | 17550188 | G | A | NM_013358:p.D116N | missense | 0.235 |  |  | O | Possibly damaging |
| MAP7D1 | chr1 | 36636667 | CC | - | NM_001286365:p.P48fs | frameshift deletion | 0.259 |  |  |  |  |
| MAP7D1 | chr1 | 36641877 | C | T | NM_001286365:p.R273W | missense | 0.145 |  |  | O | Probably damaging |
| L1TD1 | chr1 | 62676157 | A | T | NM_019079:p.K571X | nonsense | 0.207 |  |  |  |  |
| ST6GALNAC5 | chr1 | 77334341 | G | A | NM_030965:p.A59T | missense | 0.199 |  |  |  | Benign |
| PSME4 | chr2 | 54112903 | C | A | NM_014614:p.W1580L | missense | 0.083 |  |  |  | Benign |
| ZNF804A | chr2 | 185798409 | A | G | NM_194250:p.K112R | missense | 0.191 |  |  |  | Possibly damaging |
| HTT | chr4 | 3146883 | G | A | NM_002111:p.G1024E | missense | 0.097 |  |  |  | Probably damaging |
| PDE4D | chr5 | 59189292 | G | A | NM_001104631:p.P53L | missense | 0.208 |  |  |  | Benign |
| PCDHA8 | chr5 | 140222333 | C | T | NM_018911:p.T476M | missense | 0.129 |  |  |  | Probably damaging |
| TINAG | chr6 | 54173667 | G | C | NM_014464:p.E107Q | missense | 0.167 |  |  |  | Benign |
| LAMA2 | chr6 | 129588357 | A | G | NM_000426:p.E772G | missense | 0.101 |  |  |  | Benign |
| FSCN1 | chr7 | 5643631 | G | C | NM_003088:p.E417Q | missense | 0.144 |  |  |  | Benign |
| DNAH11 | chr7 | 21646161 | G | A |  | splicing | 0.146 |  |  | O |  |
| CSMD1 | chr8 | 2966208 | G | A | NM_033225:p.T2224M | missense | 0.092 |  |  | O | Probably damaging |
| TEX15 | chr8 | 30706107 | C | T | NM_031271:p.V143I | missense | 0.169 |  |  |  | Benign |
| ST18 | chr8 | 53085104 | G | C | NM_014682:p.S106X | nonsense | 0.128 |  |  |  |  |
| MRGPRX3 | chr11 | 18159561 | T | A | NM_054031:p.I271N | missense | 0.185 |  |  |  | Probably damaging |
| OSBP | chr11 | 59377890 | C | T | NM_002556:p.A179T | missense | 0.061 |  |  |  | Probably damaging |
| PTPRO | chr12 | 15637088 | G | C | NM_002848:p.A86P | missense | 0.085 |  |  |  | Probably damaging |
| GLI1 | chr12 | 57865281 | G | A | NM_001160045:p.A792T | missense | 0.17 |  |  |  | Benign |
| E2F7 | chr12 | 77419735 | G | A | NM_203394:p.S723F | missense | 0.276 |  |  |  | Possibly damaging |
| HIP1R | chr12 | 123342746 | C | T | NM_003959:p.A638V | missense | 0.05 |  |  |  | Probably damaging |
| DYNC1H1 | chr14 | 102514895 | G | A | NM_001376:p.A4421T | missense | 0.071 |  |  |  | Benign |
| OR3A3 | chr17 | 3324334 | G | T | NM_012373:p.W158L | missense | 0.087 |  |  |  | Benign |
| NF1 | chr17 | 29679366 | C | T | NM_000267:p.R2496X | nonsense | 0.176 | O | Pathogenic | O |  |
| EPG5 | chr18 | 43440188 | C | T | NM_020964:p.G2297D | missense | 0.035 |  |  |  | Benign |
| TNFSF14 | chr19 | 6670065 | C | T | NM_003807:p.V6I | missense | 0.169 |  |  |  | Benign |
| ANKLE1 | chr19 | 17397477 | G | T | NM_001278444:p.V637L | missense | 0.171 |  |  |  |  |
| ARFGEF2 | chr20 | 47615014 | G | A | NM_006420:p.W1135X | nonsense | 0.376 |  |  |  |  |
| ARFGEF2 | chr20 | 47615028 | G | T | NM_006420:p.D1140Y | missense | 0.373 |  |  |  | Probably damaging |
| PCK1 | chr20 | 56138753 | G | A | NM_002591:p.D311N | missense | 0.099 |  |  | O | Probably damaging |
| ZNF512B | chr20 | 62595032 | C | T | NM_020713:p.R546Q | missense | 0.121 |  |  |  | Possibly damaging |
| DSCAM | chr21 | 41414427 | C | A | NM_001271534:p.E1853X | nonsense | 0.114 |  |  | O |  |
| ABCG1 | chr21 | 43711668 | G | A | NM_004915:p.A531T | missense | 0.157 |  |  |  | Benign |
| KIAA1210 | chrX | 118223376 | G | C | NM_020721:p.T606S | missense | 0.311 |  |  |  | Possibly damaging |

*Variant allele frequency (VAF)

** Genes listed in The Cancer Gene Census tier 1 curated by COSMIC project

*** Annotation was based on the PolyPhen-2 prediction score (Probably damaging (>=0.957), possibly damaging (0.453<=score<=0.956), or benign (<=0.452))

Supplementary Table S3. Copy number alterations

| Chr | Cytoband | Start | End | Length | Event | Probe  Median | Cancer gene census* | Known DDLPS  driver genes** |
| --- | --- | --- | --- | --- | --- | --- | --- | --- |
| chr1 | q32.1 | 204,281,559 | 206,005,636 | 1,724,077 | Copy Gain | 0.33 | *MDM4, ELK4, SLC45A3* |  |
| chr2 | p11.2 | 89,864,528 | 89,879,731 | 15,203 | Copy Loss | -0.45 |  |  |
| chr4 | p11 - q11 | 49,090,052 | 50,400,000 | 1,309,948 | Copy Loss | -0.36 |  |  |
| chr5 | p13.3 | 32,221,894 | 32,689,022 | 467,128 | Copy Gain | 0.30 |  |  |
| chr7 | p22.3 | 935,029 | 2,692,658 | 1,757,629 | Copy Gain | 0.45 |  |  |
| chr7 | p22.1 | 5,336,743 | 5,832,678 | 495,935 | Amplification | 1.04 |  |  |
| chr7 | p22.1 | 5,832,678 | 7,274,110 | 1,441,432 | Copy Gain | 0.38 | *PMS2, RAC1* |  |
| chr7 | p21.2 - p15.2 | 14,887,777 | 25,675,201 | 10,787,424 | Copy Loss | -0.29 |  |  |
| chr7 | p15.1 - p14.3 | 28,256,916 | 30,092,491 | 1,835,575 | Copy Loss | -0.33 |  |  |
| chr8 | q11.1 - q11.21 | 46,925,881 | 51,314,792 | 4,388,911 | Amplification | 2.58 |  |  |
| chr8 | q12.1 | 56,273,403 | 56,537,748 | 264,345 | Amplification | 1.42 |  |  |
| chr9 | q31.1 - q31.2 | 106,582,659 | 108,287,186 | 1,704,527 | Copy Gain | 0.56 |  |  |
| chr9 | q31.2 - q31.3 | 109,773,075 | 113,496,557 | 3,723,482 | Copy Gain | 0.58 | *KLF4* |  |
| chr9 | q31.3 | 113,496,557 | 114,124,348 | 627,791 | Amplification | 3.06 |  |  |
| chr9 | q31.3 - q32 | 114,124,348 | 117,651,055 | 3,526,707 | Copy Gain | 0.64 |  |  |
| chr12 | q13.11 | 47,407,591 | 48,238,454 | 830,863 | Amplification | 1.90 |  |  |
| chr12 | q13.12 | 49,878,337 | 50,045,797 | 167,460 | Copy Gain | 0.48 |  |  |
| chr12 | q13.13 | 51,693,049 | 52,115,523 | 422,474 | Amplification | 1.43 | *ATF1* |  |
| chr12 | q13.2 | 54,930,769 | 56,322,764 | 1,391,995 | Amplification | 1.84 | *HOXC13* |  |
| chr12 | q13.2 | 56,374,614 | 56,415,214 | 40,600 | Amplification | 1.98 |  |  |
| chr12 | q13.3 | 57,911,218 | 58,030,117 | 118,899 | Amplification | 2.42 |  | *DDIT3* |
| chr12 | q14.1 | 58,140,453 | 58,856,195 | 715,742 | Amplification | 2.60 | *CDK4* | *CDK4* |
| chr12 | q14.1 | 61,301,180 | 62,698,190 | 1,397,010 | Amplification | 2.04 |  |  |
| chr12 | q15 | 69,036,504 | 71,471,798 | 2,435,294 | Amplification | 2.80 | *PTPRB* | *MDM2, YEAST4, FRS2* |
| chr12 | q15 - q21.1 | 71,471,798 | 73,015,178 | 1,543,380 | Copy Gain | 0.54 |  |  |
| chr12 | q21.1 | 73,015,178 | 75,400,351 | 2,385,173 | Amplification | 2.78 |  |  |
| chr12 | q21.1 - q21.2 | 75,400,351 | 77,158,050 | 1,757,699 | Copy Gain | 0.40 |  |  |
| chr14 | q12 | 31,221,384 | 31,487,674 | 266,290 | Amplification | 2.78 |  |  |
| chr19 | p12 | 22,061,088 | 23,639,115 | 1,578,027 | Copy Gain | 0.58 |  |  |
| chr19 | q13.43 | 59,028,338 | 59,128,983 | 100,645 | Copy Gain | 0.30 |  |  |
| chr20 | q12 - q13.12 | 39,317,391 | 42,788,432 | 3,471,041 | Copy Gain | 0.44 | *MAFB, TOP1, PLCG1, PTPRT* |  |
| chr20 | q13.12 - q13.13 | 45,575,209 | 46,980,996 | 1,405,787 | Copy Gain | 0.52 |  |  |
| chr20 | q13.13 | 47,610,893 | 48,600,300 | 989,407 | Copy Gain | 0.51 |  |  |
| chr20 | q13.2 | 50,363,371 | 51,053,842 | 690,471 | Copy Loss | -0.26 | *SALL4* |  |
| chr20 | q13.33 | 58,806,907 | 59,870,779 | 1,063,872 | Copy Gain | 0.38 |  |  |

* Genes listed in The Cancer Gene Census tier 1 curated by COSMIC project

** Dedifferentiated liposarcoma (DDLPS)


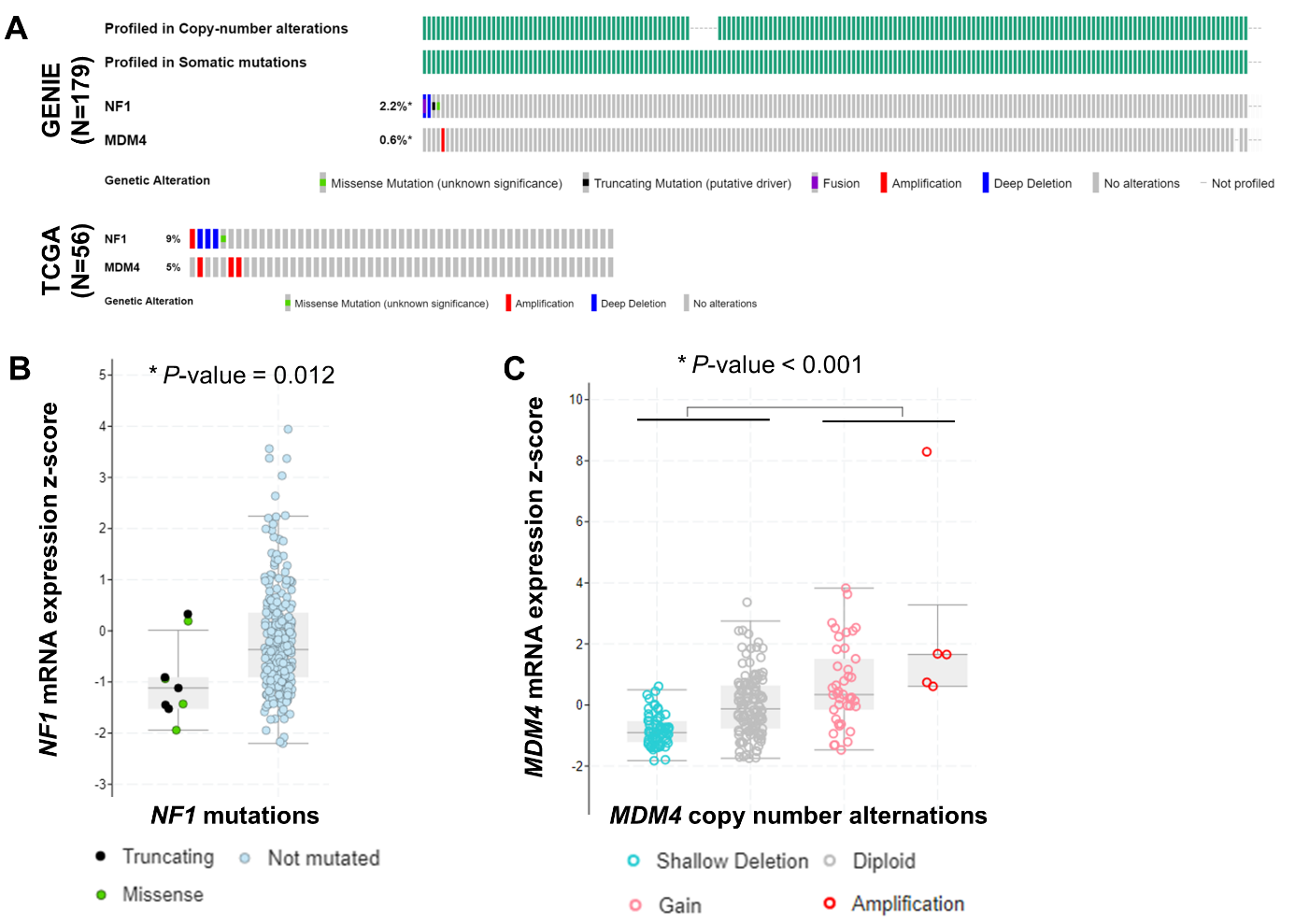


Figure S1. Analysis of *NF1* and *MDM4* alternations in open source database. (A) *NF1* and *MDM4* genetic alternations in DDLPS cases of GENIE (N=179) and TCGA (N=56). (B) *NF1* mRNA expression according to *NF1* mutation status in TCGA soft tissue sarcoma study. Each dot represents each case, and color of dot represents type of mutation. *P*-values are calculated by Mann-Whitney U test. (C) *MDM4* mRNA expression according to *MDM4* copy number alternation status in TCGA soft tissue sarcoma study. Each dot represents each case, and color of dot represents type of copy number alternation. *P*-values are calculated by Mann-Whitney U test.


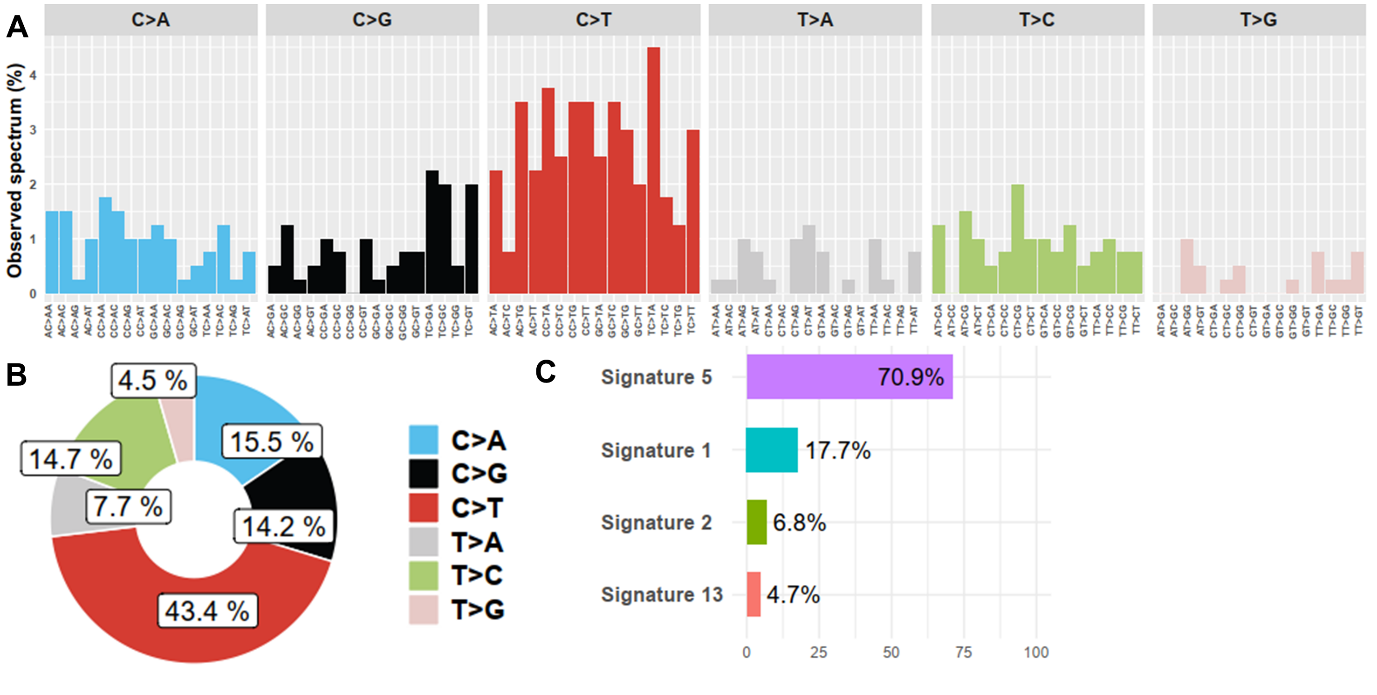


Figure S2. Mutational signature analysis. (A) Bar charts illustrate mutational signatures by disease types according to the 96-trinucleotide mutational context. (B) Pie charts of base pair alternation composition. (C) Bar charts of the result of mutational signature decomposition
